# Cytotoxic Effect of *Coscinium fenestratum* on Human Head and Neck Cancer Cell Line (HN31)

**DOI:** 10.1155/2015/701939

**Published:** 2015-05-17

**Authors:** Saranyapin Potikanond, Natthakarn Chiranthanut, Parirat Khonsung, Supanimit Teekachunhatean

**Affiliations:** ^1^Department of Pharmacology, Faculty of Medicine, Chiang Mai University, Chiang Mai 50200, Thailand; ^2^Center of Thai Traditional and Complementary Medicine, Faculty of Medicine, Chiang Mai University, Chiang Mai 50200, Thailand

## Abstract

*Coscinium fenestratum* is widely used as a medicinal plant in many Asian countries. This study aimed to investigate the cytotoxic effect of a crude water extract of *C. fenestratum* (CF extract) compared to 5-fluorouracil (5-FU) on human HN31 cell line, a metastatic squamous cell carcinoma of the pharynx. The results revealed that cell morphology visualized under inverted light microscopy was changed from flat with a polygonal appearance to round appearance after CF extract application. The cell viability assay (MTT test) showed that the concentration producing 50% growth inhibition (IC_50_) at 48-hour incubation of CF extract on HN31 was 0.12 mg/mL, while the IC_50_ of 5-FU was 6.6 mg/mL, indicating that CF extract has a higher potency. However, combining various concentrations of 5-FU and CF extract at IC_50_ did not show synergistic effect. The CF extract dose dependently increased cell apoptosis determined by Annexin-V and propidium iodide staining. It decreased the phosphorylation of p38 MAPK and pAkt, while it increased the tumor suppressor protein p53. In conclusion, the cytotoxicity of CF extract was associated with the modulation of p38 MAPK, pAkt, and p53 signal molecules, leading to inhibiting cell survival and increasing apoptosis. No synergistic effects of CF extract and 5-FU were observed.

## 1. Introduction

Head and neck cancer is defined as cancer that arises in the head or neck regions including the nasal cavity, oral cavity, larynx, and pharynx. Most head and neck cancers are squamous cell carcinomas. Although various treatment options are available, including chemotherapeutic agents such as 5-fluorouracil (5-FU) and cisplatin, those agents have not dramatically improved overall five-year survival rates [1–3]. Two main reasons for that lack of improvement are resistance to classical cytotoxic agents [4] and rapid lymphatic dissemination and metastasis, both of which contribute to the poor prognosis [5]. Traditional anticancer agents, prepared mainly from natural herbs, represent a potentially effective alternative.

*Coscinium fenestratum, *or yellow vine, in the family Menispermaceae, a woody climber with yellow wood and sap, is widely used as a medicinal plant in many Southeast Asian countries [6–8] for fever, muscle pain, abdominal pain, inflammation [9], and malaria [10]. In addition,* C. fenestratum* has been reported to possess antioxidant [11], hypotensive [7, 12], antidiabetic [13], lipid lowering [14], antiplasmodial [10], and antibacterial [15] activities. Phytochemical studies have shown that the main alkaloidal constituent of* C. fenestratum* is berberine [16, 17] in addition to a smaller amount of protoberberine [6, 18]. The antineoplastic effects of berberine have been demonstrated in several studies [19, 20]. Berberine has been shown to inhibit the growth of tumor cell lines including cell lines of breast cancer [21], melanoma [22], liver cancer, and pancreatic cancer [23]. Moreover, berberine has been shown to have an antimutagenic effect on animal models [24] and to decrease the invasive properties of various tumors [25–35]. Other studies have demonstrated that berberine obtained from* C. fenestratum* has an antiproliferative effect on human nonsmall cell lung adenocarcinoma (NCI-H838 cell line) [36], colorectal cancer cells [37], and acute myeloid leukemia (HL-60 cell line) [38]. Different cancer cells respond differently to* C. fenestratum* [39], but the effect of* C. fenestratum* on head and neck squamous cell carcinoma (HNSCC) has not yet been investigated. The objective of this study was to investigate the cytotoxic effect of a crude water extract of* C. fenestratum *compared to 5-FU on the human HNSCC cell line, HN31.

## 2. Materials and Methods

### 2.1. *C. fenestratum* Extract and 5-FU

The* C. fenestratum* used in this study was identified by the Faculty of Pharmacy and collected by the Faculty of Medicine, Chiang Mai University (voucher number PHCO-CM 028) [12]. The specimens were prepared from 100 g of dried* C. fenestratum* stems cut into thin pieces which were boiled in 500 mL of distilled water and then filtered. The procedure was repeated three times and the water extract was pooled and concentrated using a rotary evaporator at 70°C and then finally spray-dried yielding a total of 10 g of* C. fenestratum* extract (CF extract). The dried powder extract obtained was stored in a desiccator (25°C) until being used. The CF extract used in the experiment was freshly prepared by dissolving it in either distilled water or cell culture medium [12]. The 5-FU (or EFFCIL) used as a positive control for comparison of the cytotoxic effect with CF extract was obtained from Boryung Pharmaceutical Co. Ltd., Korea.

### 2.2. Cell Culture

The HNSCC cell line HN31 was kindly provided by Associate Professor Dr. Prasit Pavasat (Faculty of Dentistry, Chulalongkorn University, Bangkok, Thailand) [40]. HN31 is a metastatic lymph node squamous cell carcinoma of the pharynx [41]. Cells were maintained in Dulbecco's Modified Eagle's Medium (DMEM) with L-glutamine (Gibco, UK) supplemented with 10% fetal bovine serum (FBS), (Gibco, UK), 100 IU/mL penicillin, and 100 mg/mL streptomycin. The cells were cultured at 37°C in a humidified atmosphere with 5% CO_2_. Cells were detached from the culture flask by treatment with 0.02% EDTA or 0.25% trypsin for 10 min. at 37°C and then subcultured when 70–80% confluence was reached, approximately every 2-3 days.

### 2.3. Cell Morphological Determination

Cells were incubated in either the absence or the presence of CF extract in serum-free medium for 48 h in 24-well plates. Morphology of cells was visualized using a light inverted microscope at 400x magnification and a digital camera.

### 2.4. Cell Viability Assay

Assay of the cytotoxic effect of the CF extract was performed using MTT dye (a yellow power of 3-(4,5-dimethylthiazol-2-yl)-2,5-diphenyl-tetrazolium bromide) [40]. This colorimetric assay is a standard test for assessing cell viability. Living cells have NAD(P)H-dependent cellular oxidoreductase enzymes which reduce the MTT dye to insoluble formazan. Cells (1 × 10^4^ cells/well) were incubated on 96-well tissue culture plates in serum-free medium overnight. Different concentrations (0.01, 0.1, 1, 2.5, 5, 10, and 25 mg/mL) of either CF extract or 5-FU were applied for 48 h. Following that, the medium was discarded and the cells were incubated with MTT solution for 4 h at 37°C and under 5% CO_2_. The formazan crystals were solubilized by incubating the cells with 100% dimethyl sulfoxide (DMSO) and were immediately measured at a wavelength of 540 nm using a microplate reader. The concentration at which 50% cell growth inhibition occurred (IC_50_) was calculated using a nonlinear curve fit tool (GraphPad Prism version 5). To determine whether a synergistic effect exists between 5-FU and CF extract, the cell lines were incubated with a mixture of CF extract at its IC_50_ and various concentrations of 5-FU for 48 h under the same condition as aforementioned.

### 2.5. Determination of Apoptosis

The effect of CF extract on cell apoptosis was determined using an Annexin-V-FITC and propidium iodide kit (Invitrogen). Living cells which have intact plasma membranes are not stained by Annexin-V-FITC or propidium iodide fluorescent dye. However, when apoptosis occurs, phospholipid phosphatidylserine [42] is translocated from the inner to the outer leaflet and will be exposed and stained with Annexin-V-FITC. When cells are dead, DNA can be easily labeled with propidium iodide. Tests were accomplished according to the protocol provided by the manufacturer. 3 × 10^5^ cells in 24-well plates were starved in serum-free medium (SFM) overnight [43]. Then, either CF extract or 5-FU at different concentrations was added. After 18 h incubation, cells were trypsinized and washed two times in phosphate buffered saline (PBS). Binding buffer followed by Annexin-V-FITC and propidium iodide were applied in the dark for 10 minutes. The apoptotic and surviving fractions were determined by flow cytometry.

### 2.6. Gel Electrophoresis and Immunoblot Assays

Protein analysis by western blotting was used to explore various cellular mechanisms. Cells were incubated either with or without CF extract for 1 h. Proteins were extracted by lysis buffer (20 mM Tris-HCl, 1 mM Na3VO4, 5 mM NaF, and a cocktail of protease and phosphatase inhibitors) and extracted in 2x sodium dodecyl sulfate polyacrylamide gel electrophoresis (SDS-PAGE) sample buffer. The protein preparations were separated using 10% SDS-PAGE. The separated proteins were then transferred to PVDF membranes (Immobilon-P) for 1 h at 100 voltage [44]. After blocking with 5% nonfat dry milk/TBST, primary antibodies were incubated for either 2 h at room temperature or overnight at 4°C. Primary antibodies (anti-caspase-3, anti-Bax, anti-Bcl2, and anti-actin) were purchased from Santa Cruz Biotech, USA. Phosphospecific p38 MAPK, pAkt, and p53 were obtained from Cell Signaling, USA. All primary antibodies were used at concentrations of 1 : 1,000. The secondary antibody was horseradish peroxidase: conjugated goat anti-mouse or goat anti-rabbit antibody. Protein bands were visualized using the Amersham ECL detection reagents system (GE Healthcare, UK) and developed on Hyperfilm (GE Healthcare, UK) [45].

### 2.7. Statistical Analysis

All data was expressed as means ± SEM. Comparisons were made using one-way analysis of variance (ANOVA) with post hoc test and *P* values of <0.05 were regarded as statistically significant.

## 3. Results

### 3.1. Cytotoxic Effect of CF Extract on Cell Morphology

**Figure 1:**
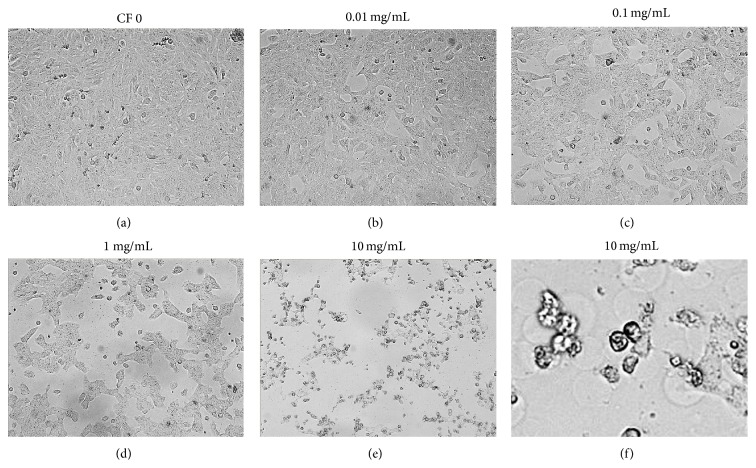
Morphology of HN31 after treatment with CF extract at 0 mg/mL (a), 0.01 mg/mL (b), 0.1 mg/mL (c), 1 mg/mL (d), 10 mg/mL (e), and 10 mg/mL at higher magnification (f).

Normal HN31 is flat with a polygonal appearance (Figure 1(a)). After CF extract (at concentrations of 1 and 10 mg/mL) was applied for 48 h, the morphological changes observed included shrinkage and a shift to a rounded appearance (Figures 1(d)–1(f)).

### 3.2. Cytotoxic Effect of CF Extract on Cell Viability

**Figure 2:**
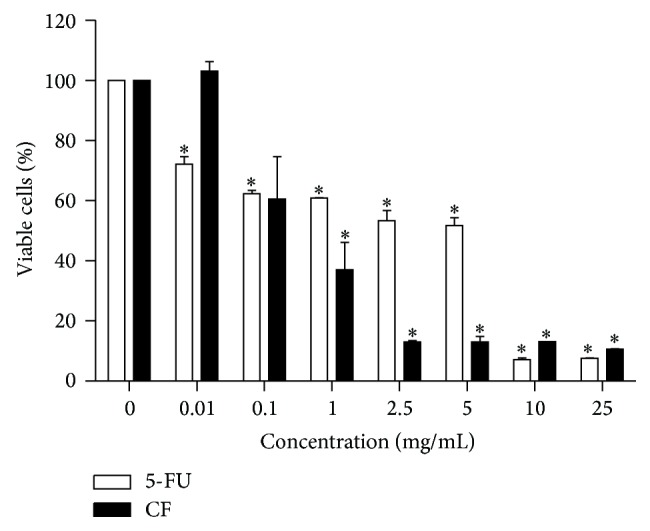
Effect of CF extract and 5-FU on cell viability. HN31 cells were incubated with different concentrations of CF extract or 5-FU for 48 h at 37°C and 5% CO_2_. Viability assay was done by MTT test. Values are means ± SEM (*n* = 4 in triplicate). ^∗^Statistically significant difference from the control (0 mg/mL).

MTT assay was used to evaluate the cytotoxic effect as nonviable cells cannot reduce or convert the MTT dye (tetrazolium dye) into insoluble formazan crystals. The intensity of the purple color was used to determine the ratio of viable cells. CF extract at a concentration of 1 mg/mL or higher significantly reduced the percentage of viable cells, while 5-FU showed significant reductions at all tested concentrations (Figure 2). Maximal effect with CF extract (approximately 80% reduction in dye intensity of control cultures) was achieved at 2.5 mg/mL, while increasing concentrations of 5-FU resulted in a more gradual decline, reaching maximal effect at 10 mg/mL. The IC_50_ at 48-hour incubation of CF extract on HN31 was 0.12 mg/mL, while the IC_50_ of 5-FU was 6.6 mg/mL, indicating that CF extract has a higher potency.

### 3.3. Combined Effect of CF Extract and 5-FU on Cell Viability

**Figure 3:**
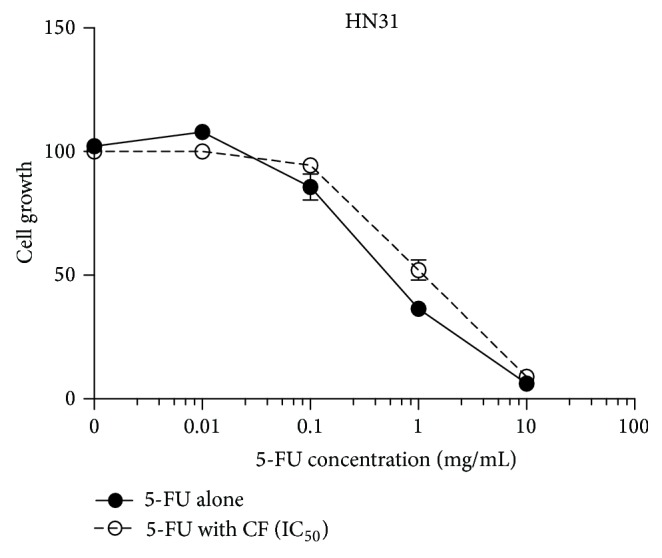
Effect on HN31 cell viability following application of different concentrations of 5-FU (5-FU alone) versus combination of CF extract at IC_50_ and the respective concentrations of 5-FU (5-FU with CF) for 48 h at 37°C and 5% CO_2_. Viability assay was done by MTT test. Values are means ± SEM (*n* = 4 in triplicate).

The dose of CF extract at IC_50_ was applied to different concentrations of 5-FU, and then cell viability MTT assay was performed. The combination of CF extract and 5-FU in varying concentrations did not show significant reductions, indicating that CF extract and 5-FU do not have a synergistic effect (Figure 3).

### 3.4. The Effect of CF Extract on Cell Apoptosis

**Figure 4:**
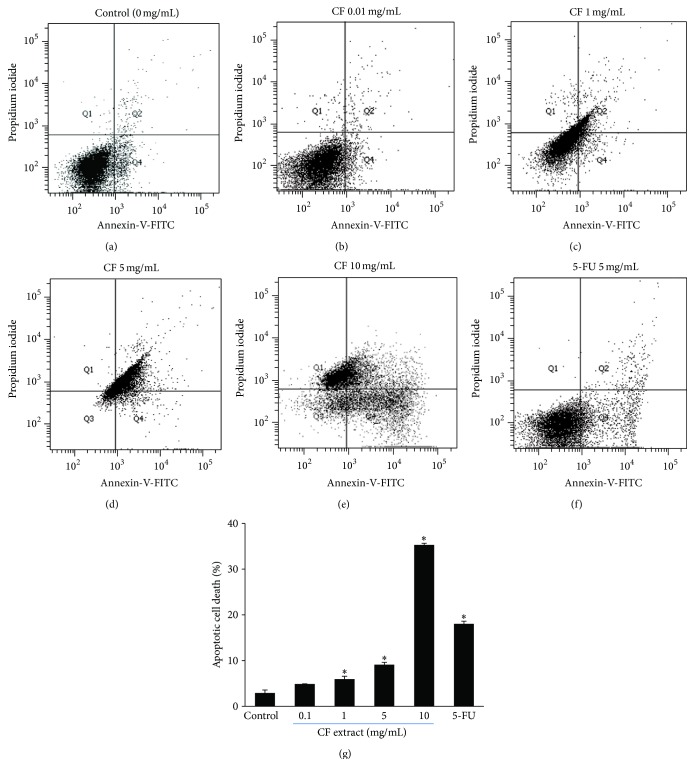
Effect of CF extract and 5-FU on HN31 cell death. Data are control (a), CF extract at 0.1 mg/mL (b), 1 mg/mL (c), 5 mg/mL (d), 10 mg/mL (e), and 5-FU at 5 mg/mL (f). The bar graph (g) shows percentage of apoptotic cell death (percentage of cells in 4th quadrant or Q4). Values in bar graph are means ± SEM (*n* = 3 in duplicate). ^∗^Significantly different from the control.

Apoptosis evaluation was performed using Annexin-V and propidium iodide assay followed by flow cytometry. Results of that assay, shown in Figure 4, indicate the number of viable, apoptotic, and necrotic cells calculated based on the presence of phosphatidylserine (PS) molecules on the apoptotic cells. Viable cells with intact membranes and unexposed PS molecules remain unlabeled (cells in the 3rd quadrant or Q3). Propidium iodide enters the compromised membranes of necrotic cells and stains the DNA (cells in the 1st quadrant or Q1). Early apoptotic cells, with exposed PS molecules but without membrane abruption, are bound with Annexin-V (cells in the 4th quadrant or Q4). The cells in the 2nd quadrant (or Q2) are early necrosis, binding with both Annexin-V and propidium iodide. After 18 h of incubation, untreated cells (control) had low levels of apoptotic cell death, determined from the percentage of cells in Q4 (2.93 ± 0.64%). The CF extract at 1, 5, and 10 mg/mL significantly increased the percentage of apoptotic cell death in comparison to the control: 5.97 ± 0.61%, 9.1 ± 0.52, and 35.30 ± 0.35%, respectively. Similarly, the percentage of apoptotic cell death following application of 5-FU at 5 mg/mL was 18.03 ± 0.61% and was also significantly greater than the control (Figure 4(g)).

### 3.5. The Effect of CF Extract on Signaling Molecules

**Figure 5:**
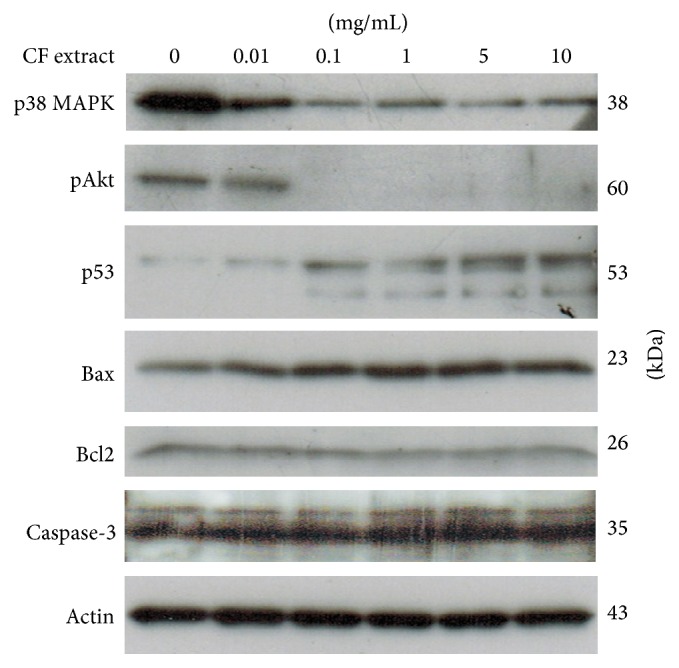
Effect of CF extract on the signaling molecules p38 MAPK, pAkt, p53, Bax, Bcl2, and caspase-3 protein in HN31 cell line, demonstrated on western blotting. Loading control was determined by actin protein.

Growth inhibition of CF extract on the HN31 cell line was found to be associated with the activation of p38 mitogen-activated protein kinases (p38 MAPK) which regulates cell proliferation, differentiation, and apoptosis. The CF extract decreased the phosphorylation of p38 MAPK in a dose-dependent fashion but slightly increased the proapoptotic protein Bax (Figure 5). The tumor suppressor molecule, p53, was dose dependently increased by the CF extract, while the tumor survival molecule, pAkt, was decreased. These results indicate that a cytotoxic effect of CF extract is likely to be mediated at least through inhibiting survival signal molecules and increasing apoptotic proteins.

## 4. Discussion

Head and neck cancer cells are among the more aggressive tumors and are less sensitive to available anticancer agents. Drugs resistance [4] and metastasis [5] are major problems. This study choses to focus on HN31 because pharyngeal cancers are among the ten most common cancers in Thailand [46] and HN31 is a particularly aggressive lymphatic metastatic cell line [41], which often fails to respond adequately to conventional chemotherapy.

Several studies have shown that the stem of* C. fenestratum* has an antiproliferative effect against many cancers [37, 39]. Since the stems of* C. fenestratum* boiled in water have long been used in Thai traditional medicine, this research processed a water extract of* C. fenestratum* to investigate its cytotoxic effect on the HN31 cell line. This study found that CF extract resulted in a reduction in cell viability and increase in apoptosis in the HN31 cell line, but no synergistic effects of CF extract and 5-FU were observed. The cytotoxic effect was mediated via modulation of p38 MAPK, pAkt, and p53 proteins.

5-FU and crude CF extract investigated in this study had cytotoxic effect, and the dose at which 50% cell growth inhibition (IC_50_) occurs is a measure of the strength or potency of a given compound: the higher the IC_50_, the lower the potency. The IC_50_ of 5-FU for the HN31 cell line reported in the present study was 6.6 mg/mL, which is relatively high, presumably due to its mutation of the tumor suppressor gene p53 [41, 47, 48]. In contrast, our preliminary study found that the IC_50_ values of 5-FU for the less invasive HNSCC cell lines were substantially lower, for example, 10.26 *μ*g/mL for HN30 and 24.98 *μ*g/mL for HN22 (unpublished data), indicating the considerably decreased susceptibility of the HN31 cell line to 5-FU. This study also found that CF extract has a higher potency on HN31 (IC_50_ of 0.12 mg/mL) than 5-FU. Other studies, however, have reported that the IC_50_ of CF extract on lung-related tumor cells was about 0.001 mg/mL [39], that is, 100 times more potent. That difference in potency may be due to the HNSCC cell line being less susceptible to CF extract than lung cancer cells or to the fact that the lung cancer study used a methanol-water CF extract which may have had more of the active ingredient berberine [17]. The dry weight of berberine obtained from alcohol crude extracts in that study was 11.84–18.45% [17]. The yield of berberine from water CF extract used in this study as well as the cytotoxicity of berberine on HN31 should be investigated further.

The present study demonstrates that CF extract can decrease the survival and proliferation of the signal molecule pAkt which is key to 5-FU chemoresistance in squamous carcinoma cells [49]. In addition to the pAkt survival pathways, the mitogen-activated protein kinases (MAPKs) are also involved in cancer chemoresistance [50]. Most HNSCC exhibits activation of p38 MAPK [51]; therefore, the inhibitory effect of CF extract on pAkt and p38 MAPK activity possibly contributes to its efficacy in preventing or reducing cancer chemoresistance. Furthermore, it has been demonstrated that p53 is another crucial protein that plays an important role in tumor suppression and tumorigenesis [48]. Therefore, if CF extract can increase the expression of p53, it could be expected that its mechanism of action is also mediated via increased tumor suppression.

Combination in cancer therapy has been standard care aiming at maximizing efficacy while minimizing systemic toxicity [52]. Each of the drugs carries its own risks and benefits. For example, 5-FU has many side effects such as myelosuppression and cardiotoxicity [53–55]. Previous research by the authors found that high doses of CF water extract did not have a toxic effect or significantly change any other parameters in rats [12]. Another study demonstrated that CF alcoholic extract does have a neurotoxic effect on rats [56]. This discrepancy might be due to the difference in the extraction solutions used. The maximal anticancer efficacy of CF extract and 5-FU used individually was comparable; however, using crude CF extract and 5-FU in combination did not have a synergistic effect. In general, the principles of combination therapy for cancer are primarily based on using drug with nonoverlapping toxicities and combining agents with different mechanism of action [52]. The reason for the lack of synergism was not immediately evident; it is postulated that the two substances might possess the similar mechanism of action or exert the similar modulation on signaling molecules of HN31. This experimental result argues against the use of a combination of crude CF extract with 5-FU in a clinical setting.

Some limitations regarding this study should be addressed. First, the active ingredient(s) of CF extract (e.g., berberine and others) have not been quantified. Second, the cytotoxic effect of CF extract on other HNSCC cell lines has not been studied. Third, the effect of CF extract on other signaling molecules (e.g., NF-kappaB signaling, PI3K/mTOR, and NOTCH, intercellular adhesion molecule-1 (ICAM-1)) involved in tumorigenesis and metastasis [57–59] has not been fully understood and warrants further investigation.

## 5. Conclusions

CF extract has potential as chemotherapeutic agent. Its cytotoxicity was associated with the modulation of p38 MAPK, pAkt, and p53 signal molecules, leading to inhibiting cell survival and increasing apoptosis. No synergistic effects of CF extract and 5-FU were observed.
